# Identifying Key Factors in University Students’ Physical and Mental Health: An Integrated Regression and Machine Learning Approach

**DOI:** 10.3390/bs16040486

**Published:** 2026-03-25

**Authors:** Qin Jiang, Sirui Wu, Nengzhong Xie, Yanxue Zhao, Yan Li, Xiaoyu Wu, Xuebing Wang

**Affiliations:** 1 School of Marxism, Guangxi University, Nanning 530004, China; 15023325765@163.com (S.W.); 3336810@gmail.com (Y.Z.); 17622738033@163.com (Y.L.); 2 Student Affairs Office, Guangxi University, Nanning 530004, China; 3 School of Physical Education, Guangxi University, Nanning 530004, China; wuxiaoyu-3@163.com (X.W.); wangxuebing@gxu.edu.cn (X.W.)

**Keywords:** physical health, mental health, correlative factors, machine learning

## Abstract

This study sought to identify key factors in university students’ physical and mental health by using a combination of methods, including classical statistical analysis and machine learning techniques. Physical and mental health test data were collected from undergraduates of the 2020 to 2023 cohorts at a university. A self-designed questionnaire on factors associated with physical and mental health was also sent to randomly selected undergraduate students from the same university. The study data were analyzed by one-way analysis of variance, Pearson correlation analysis, hierarchical regression analysis, and a machine learning model. The results revealed that participation in school sports clubs (*β* = −0.111, *p* < 0.001) and amount of exercise (*β* = 0.182, *p* < 0.001) were significant predictors of physical health status. Difficult family economic situation (*β* = 0.162, *p* < 0.001), major satisfaction (*β* = −0.092, *p* = 0.02), the quality of a romantic relationship (*β* = −0.121, *p* = 0.003), the quality of interpersonal relationships (*β* = −0.157, *p* < 0.001), and an overprotective family parenting style (*β* = 0.109, *p* = 0.011) were significant predictors of mental health status. The results of regression analysis and application of the machine learning model identified that the amount of exercise, quality of interpersonal relationships, and family parenting style had consistent effects on both the physical and mental health of university students.

## 1. Introduction

In 1948, the World Health Organization defined health as “… a state of complete physical, mental and social well-being and not merely the absence of disease or infirmity” in its constitution (World Health Organization, 1948). In 1990, it revised its interpretation of health to include sound physical health, mental health, good social adaptation, and moral health (B. Sang, 2022). Bircher (2005) subsequently proposed that health is a dynamic state of well-being characterized by physical, mental, and social potential that satisfies the demands of life commensurate with age, culture, and personal responsibility. This evolution of the concept of health shows that this notion had been continuously expanding from “health equals the absence of disease” to an emphasis on the integrity of physical, mental, and social well-being. Particularly, although the understanding of the concept of health has varied across time, physical health and mental health have remained essential components of the modern concept of health.

In line with the evolving conceptualization of health, the biopsychosocial model provides a crucial theoretical framework that explicitly integrates the social dimension alongside biological and psychological factors. This model, first formally proposed by psychiatrist Engel in 1977 as a challenge to the reductionist biomedical model (Engel, 1977), posits that health and illness are the result of the complex, dynamic interaction between biological, psychological, and social factors (Wade & Halligan, 2017). The biopsychosocial model is fundamentally systemic and holistic, conceptualizing the individual not merely as a biological entity but as a person situated within a social context, whose subjective experience of illness and wellness is shaped by this interplay (Borrell-Carrió et al., 2004).

The physical and mental health of university students is crucial for social development. As university students are in a critical period of biological, psychological, and social transitions, a comprehensive understanding of the factors associated with their health must be situated within the biopsychosocial model. This model posits that research must transcend a reductionist focus on isolated biological or psychological traits, and instead critically incorporate the analysis of social factors. Based on this, the present study will particularly explore how certain social factors, in interaction with biological and psychological factors, collectively associated with the physical and mental health of university students.

Biological and psychological factors do not operate in isolation but rather engage in continuous interaction. Several studies have found a correlation between physical health and mental health among university students (Muraki et al., 1993; Avitsland et al., 2020). An adverse mental health state could have a negative impact on physical health. For example, Y. Wang and Liu (2025) found that university students exhibiting moderate to severe depressive symptoms were significantly more likely to engage in health-risk behaviors, with odds 3.2 times higher for binge drinking, 4.1 times greater for tobacco use, and 2.8 times higher for physical inactivity compared to their peers with minimal symptoms. Conversely, maintaining good physical health has a positive impact on mental health. A study by Jeoung et al. (2013) examined the correlation between health-related physical fitness and mental health in university students and found that muscle strength, muscle endurance, and body weight influenced mental health. Wetzler and Ursano (1988) also found that mental health was associated with health-related behaviors, including sleep patterns, physical exercise, breakfast habits, frequency of snacking, relative body weight indicators, smoking, and alcohol consumption. These investigators proposed that such health-related behaviors may function as critical mediators in the mind–body interaction.

An individual’s health is ultimately shaped by the complex interplay of biological, psychological, and social factors. Numerous studies focusing on university students have revealed that various social factors significantly influence their physical and mental health. These factors typically exert their effects by shaping individuals’ behavioral patterns, psychological states, and living environments.

Lifestyle has been consistently highlighted in previous studies as a critical factor influencing the physical and mental health of university students (Bi et al., 2014; Melnyk et al., 2016; Fan et al., 2020). Bi et al.’s (2014) research revealed that health status in university students was significantly positively correlated with lifestyle, and for each dimension of the “Health Promoting Lifestyle Profile-II” model, including “spiritual growth”, “health responsibility”, “physical activity”, “interpersonal relations” and “stress management”, the mean scores of participants reporting “suboptimal health status” or “disease” were lower than those of participants reporting “healthy”. Research by Walsh (2011) suggested that healthy eating habits, regular physical exercise, and spending time in contact with nature promoted good mental health. Furthermore, a significant negative correlation between symptoms of depression/anxiety and healthy lifestyle beliefs/behaviors was identified by Melnyk et al. (2016).

Physical exercise is another important factor that associated with the physical and mental health of university students (Saxena et al., 2005; Walsh, 2011; Ghosh & Datta, 2012). A study by Saxena et al. (2005) found that physical exercise could reduce the incidence of common mental disorders and alleviate symptoms of depression. The study by Ghosh and Datta (2012) also found that physical exercise had a significant positive effect on emotional state, could boost self-esteem, and could reduce stress tendencies, thereby preventing physical and mental illnesses. Moreover, according to a study in adolescents by Joung et al. (2024), the enjoyment derived from participation in school sports clubs increased the likelihood of engagement in sports activities and provided a sufficient motivational foundation for sustained participation. Involvement in sports clubs might improve the overall quality of physical activity, contributing to mitigation of the health issues associated with obesity and overweight in adolescents.

Quality of sleep also affects the physical and mental health of university students (Pujitha et al., 2019; Clement-Carbonell et al., 2021). Poor sleep quality has a negative impact on physical and mental health. Stein et al. (2008) found that sleep problems often co-occurred with other physical and mental health problems. Pujitha et al. (2019) reported that physical health in university students was positively correlated with sleep quality, while. Clement-Carbonell et al. (2021) found that sleep quality was significantly associated with both physical and mental health status, with a stronger correlation observed for mental health.

Other studies had found that factors such as quality of interpersonal relationships, the quality of a romantic relationship, excessive use of electronic devices, family parenting style, and academic performance have a significant effect on the physical and mental health status of university students (Braithwaite et al., 2010; Z. Q. Sang & Fu, 2015; Segev et al., 2015; Li & Li, 2015; Shi et al., 2024; Monzonís-Carda et al., 2024). For instance, Braithwaite et al. (2010) demonstrated that university students in committed romantic relationships experienced significantly better mental health and a lower likelihood of being overweight/obese compared to their single counterparts. The study further revealed that this protective effect operates primarily through behavioral regulation, as being in a committed relationship is associated with having fewer sexual partners, which in turn leads to a reduction in other risky behaviors, thereby contributing to improved well-being. Excessive use of electronic devices, social media, or video games has been identified to have a negative impact on mental health status in university students (Segev et al., 2015; Andreassen et al., 2016). Similarly, mobile phone dependence has been associated with adverse effects on their physical health (Alotaibi et al., 2022). Monzonís-Carda et al. (2024) also proposed that academic performance could significantly influence the mental health of university students and is a predictor of their mental health status.

Previous research has used primarily correlation and regression analyses to investigate the factors associated with physical and mental health in university students. However, the ability of these methods to capture nonlinear relationships among complex factors and potential interaction effects is limited. In recent years, machine learning has demonstrated significant potential for processing and analyzing large-scale, multidimensional data. Machine learning can model complex nonlinear relationships among numerous variables and outcomes and has been widely applied by researchers in the clinical psychology and psychiatry fields. Moreover, machine learning can assess the contributions of multiple factors, enabling the identification and ranking of the most influential predictors. To date, no study has yet developed an integrated machine learning model that simultaneously predicts both the physical and mental health of university students. Therefore, the present study utilized a substantial sample size and well-established assessment methods to conduct a comprehensive evaluation of the physical and mental health status of university students. Moreover, existing research tends to focus on isolated or limited dimensions of factors, often lacking an integrated theoretical framework to systematically and simultaneously examine multidimensional factors collectively shaping the health of university students. Therefore, guided by the biopsychosocial model, the aims of this study were to identify social dimension factors that associated with the physical and mental health of university students, elucidate the underlying mechanisms, and develop a model for prediction of their physical and mental health status. By integrating classical statistical analysis methods with machine learning techniques, the present study adopted a multidimensional analytical approach to examine the complex correlative factors of student health outcomes from biopsychosocial perspectives. The findings would provide new perspectives and empirical support for developing a systematic and comprehensive theoretical framework for the physical and mental health of university students.

## 2. Materials and Methods

### 2.1. Participants

The present study analyzed the results of routine physical health tests (21,588 valid data samples) and mental health tests (20,564 valid data samples) for undergraduate students of the 2020 to 2023 cohorts attending a university in Guangxi Province, China. A self-designed questionnaire on factors associated with physical and mental health was distributed to 1600 undergraduate students via the Questionstar website (an online survey platform, https://www.wjx.cn/). Their routine physical and mental health data were matched to the responses to the questionnaire using student identification numbers. Questionnaires with missing basic information or response times of less than 60 s were excluded. A total of 1416 valid questionnaires (88.5%) were returned. All the study data were anonymized by removing student identification numbers, class information, names, and other identifying details to protect the privacy of subjects. The study was approved by the ethics committee of the affiliated university (GXU-2024-073). 

### 2.2. Measures

Routine physical health data were analyzed for all undergraduates in the 2023–2024 academic year. A higher total score indicates better physical health. A total physical health test score was categorized as follows: ≥90.0, excellent; 80.0–89.9, good; 60.0–79.9, pass; and ≤59.9, fail. Mental health data were collected in March 2024 using the University Personality Inventory (UPI), which has been widely used to assess mental health in university students (Yu et al., 2024; Y. Zhang et al., 2024; Q. Y. Wang & Xiao, 2025). This questionnaire consists of 60 items, including 4 deception-check items. A response of “yes” scores 1 point and a response of “no” scores 0 points. The total score is 56 points (excluding deception–check items). A lower score indicates better mental health and vice versa. Based on the results, the mental health status of an individual can be classified into three categories: class I, indicating potentially serious psychological problems; class II, indicating potentially general psychological problems; and class III, indicating no mental health issues. Students who meet any of the following criteria are graded as class I: a total UPI score ≥ 25 points; a response of “yes” to item 25; a response of “yes” to at least two auxiliary items; and clear consultation requirements. Students who meet any of the following criteria are graded as class II: total UPI score between 20 and 25 points; a response of “yes” to items 8, 16, and/or 26; and a response of “yes” to only one of the auxiliary items. Students that cannot be graded as class I or II are classified as class III. In this study, the Cronbach’s α coefficient for the UPI was 0.94.

The self-designed questionnaire on factors associated with physical and mental health was initially designed based on a comprehensive synthesis of literature on university student health. Individual items for key constructs (e.g., the three family parenting styles, the amount of exercise) were selected or adapted from established, validated scales, prioritizing items with demonstrated high factor loadings. This approach was taken to ensure that each single item was a robust, face-valid indicator of its intended construct. The draft questionnaire underwent iterative review and revision by a panel of experts, including frontline physical education teachers and psychological counselors, to evaluate the suitability, clarity, and relevance of each item and the overall structure. The questionnaire consisted of two parts with a total of 30 items. The first part was designed to collect demographic data, including sex, academic major, and academic year. The second part examined factors associated with physical and mental health. For physical health, categorical variables included participation in school sports clubs, Internet usage time, sleep duration, sedentary time, breakfast habits, smoking status, and alcohol consumption. Continuous variables were satisfaction with sports facilities, family/school/social exercise atmosphere, and amount of exercise. For mental health, categorical variables included place of origin, family structure, only child status, family economic situation, and academic performance. Continuous variables included major satisfaction, quality of romantic and interpersonal relationships, and family parenting style. For the items involving continuous variables in the questionnaire, individuals were required to judge whether the descriptions of the items reflected their personal circumstances. A 5-point Likert scale was used (1, “completely inconsistent”; 2, “inconsistent”; 3, “average”; 4, “consistent”; and 5, “completely consistent”).

### 2.3. Statistical Analysis

One-way analysis of variance (ANOVA) was employed to examine the relationships between categorical variables and the physical and mental health of university students. Pearson correlation analysis was used to explore the associations of continuous variables with physical and mental health. Multiple linear regression analysis was performed to establish a model of potential influencing factors. The statistical analysis was performed using SPSS 27.0 statistical software (IBM Corp., Armonk, NY, USA). A *p*-value of <0.05 was considered statistically significant.

### 2.4. Machine Learning Methods

The analysis was conducted using Python 3.9.12 within the Jupyter Notebook 7.2.1 environment. Data preprocessing, including data cleaning, feature selection, and data standardization, was performed using the Pandas 2.2.3 and NumPy libraries 2.2.4. The Scikit-learn 1.6.1, XGBoost (https://xgboost.readthedocs.io/en/stable/), and LightGBM (https://lightgbm.readthedocs.io/) libraries were used to construct the model (Sharda et al., 2025; Ruiz Pujadas et al., 2025).

For analysis of the data by machine learning, the physical health test results were classified into physically healthy (acceptable, good, or excellent) and physically un-healthy (poor). The UPI test results for mental health were divided into mentally healthy (no mental health issues) and mentally unhealthy (with potentially general or severe psychological problems). Based on the above classification results, the overall status of physical and mental health was further classified into “physically and men-tally healthy” and “physically or mentally unhealthy”. Physically and mentally healthy indicated that both physical and mental health were in the healthy category, while physically or mentally unhealthy included any of the following three combinations: physically healthy but mentally unhealthy, mentally healthy but physically un-healthy, and both physically and mentally unhealthy. While dichotomizing the physical and mental health outcomes results in a loss of information from the original, more nuanced scales, it simplifies the classification problem for machine learning. This approach aligns with a pragmatic goal of identifying university students “at risk” (i.e., unhealthy) for targeted intervention, and is supported by methodological precedents in health research that employ binary outcomes for risk prediction and screening purposes (Baba & Bunji, 2023; Bhattacharjee et al., 2025; Grim et al., 2024).

The dataset was partitioned into training and test sets at a 70:30 ratio. The Synthetic Minority Oversampling Technique (SMOTE; Chawla et al., 2002) was applied to the full training set prior to cross-validation to rebalance the class distribution, given the substantial class imbalance in the target variable (physically and mentally healthy: n = 269; physically or mentally unhealthy: n = 1144). The test set was retained in its original class distribution throughout, and final model performance was evaluated exclusively on this untouched test set to ensure unbiased evaluation. Model development was subsequently conducted using 5-fold cross-validation on the training set, with optimal hyperparameter configurations identified for each model via Grid Search (GridSearchCV).

To ensure model robustness, provide a fair comparison baseline, and increase the transparency of the results, rigorous validation procedures were implemented across all evaluated algorithms. Specifically, a 5-fold cross-validation procedure was applied to the training set during the model development phase. Hyperparameter tuning was systematically conducted using Grid Search (GridSearchCV) to identify the optimal parameter combinations for each respective model, optimizing over the following ranges and yielding the following optimal configurations:

NaiveBayes: var_smoothing {1 × 10^−9^, …, 1}; optimal: var_smoothing = 0.0001.

Decision Tree: max_depth {None, 5, 10, 20}, min_samples_leaf {1, 2, 4}, min_samples_split {2, 5, 10}; optimal: max_depth = None, min_samples_leaf = 4, min_samples_split = 10.

RandomForest: n_estimators {100, 200}, max_depth {5, 8, 10}, min_samples_leaf {4, 8, 16}; optimal: n_estimators = 200, max_depth = 10, min_samples_leaf = 4.

KNN: n_neighbors {3, 5, 7, 9}, weights {uniform, distance}; optimal: n_neighbors = 3, weights = distance.

XGBoost: n_estimators {100, 200}, max_depth {3, 5}, learning_rate {0.01, 0.1}, min_child_weight {3, 5}; optimal: n_estimators = 200, max_depth = 5, learning_rate = 0.1, min_child_weight = 3.

LightGBM: n_estimators {100, 200}, num_leaves {15, 31}, learning_rate {0.01, 0.1}, min_child_samples {20, 30}; optimal:n_estimators = 200, num_leaves = 31, learning_rate = 0.1, min_child_samples = 20.

## 3. Results

### 3.1. General Characteristics of the Survey Subjects

#### 3.1.1. Full Cohort (Overall Sample)

**Figure 1:**
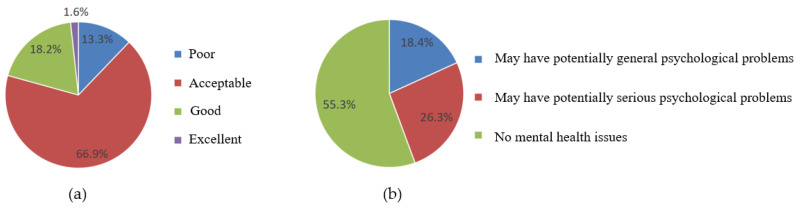
(**a**) Overall physical health status of university students; (**b**) Overall mental health status of university students.

A total of 21,588 university students underwent the physical health test during the study period. The cohort consisted of 12,215 men (56.58%) and 9373 women (43.42%). By academic major, 15,855 students (73.45%) were enrolled in science and engineering, 5081 (23.53%) in humanities and social sciences, and 652 (3.02%) in arts and physical education. By academic year, 5477 (25.37%) were freshmen, 5259 (24.36%) were sophomores, 5350 (24.79%) were juniors, and 5502 (25.47%) were seniors. According to the National Student Physical Health Standards (NSPHS, 2014 Revision, China), physical health was poor in 13.3% (n = 2878), acceptable in 66.9% (n = 14,445), good in 18.2% (n = 3918), and excellent in 1.6% (n = 347) (Figure 1, left panel).

The UPI was administered to 20,564 university students. The sample consisted of 11,087 men (53.91%) and 9477 women (46.09%). By academic major, 14,617 (71.08%) were enrolled in science and engineering, 5109 (24.84%) in humanities and social sciences, and 838 (4.08%) in arts and physical education. By academic year, 5642 (27.44%) were freshmen, 5166 (25.12%) were sophomores, 4826 (23.47%) were juniors, and 4930 (23.97%) were seniors. Based on UPI scores, 11,363 (55.3%) were classified as no mental health issues, while 3789 (18.4%) and 5412 (26.3%) showed indications of potentially general and serious psychological problems, respectively (Figure 1, right panel).

#### 3.1.2. Analytical Subsample (Questionnaire Respondents)

A subsample of 1416 university students completed the full assessment, including the physical health test, the mental health test, and the questionnaire on factors associated with their physical and mental health. There were 762 men (53.81%) and 654 women (46.19%); 1252 were majoring in science and engineering (88.42%), 156 in humanities and social sciences (11.02%), and 8 in arts and physical education (0.56%). There were 618 freshmen (43.64%), 437 sophomores (30.86%), 347 juniors (24.51%), and 14 seniors (0.99%).

**Figure 2:**
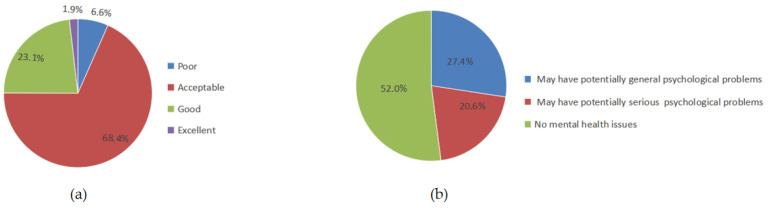
(**a**) Physical health status of the subsample; (**b**) Mental health status of the subsample.

According to the NSPHS, the physical health status of this subsample was rated as poor in 6.6% (n = 94), acceptable in 68.4% (n = 969), good in 23.1% (n = 327), and excellent in 1.9% (n = 26) (Figure 2, left panel). Based on UPI scores, 737 (52.0%) were classified as no mental health issues, while 388 (27.4%) and 291 (20.6%) showed indications of potentially general and serious psychological problems, respectively (Figure 2, right panel). The statistical and machine learning analyses of factors associated with the physical and mental health of these students were performed on this part of the data alone.

### 3.2. Comparison of Physical and Mental Health Status Among University Student Groups with Different Characteristics

#### 3.2.1. Comparison of Physical Health Status Among University Student Groups with Different Characteristic

One- way ANOVA was performed to compare the total physical health scores across groups defined by categorical variables. These variables, derived from the self-designed questionnaire, included participation in school sports clubs, Internet usage time, sleep duration, sedentary time, breakfast habits, smoking status, and alcohol consumption. The total physical health test scores varied significantly according to participation in school sports clubs (*F*(1, 1414) = 15.349, *p* < 0.001, *η*^2^ = 0.011), sedentary time (*F*(3, 1412) = 3.090, *p* = 0.026, *η*^2^ = 0.007), and breakfast habits (*F*(3, 1412) = 4.916, *p* = 0.002, *η*^2^ = 0.010). Post hoc analysis showed that the total physical health score was significantly higher in students who participated in a school sports club than in those who did not, in students who sat for 3–4 h or for longer than 5 h than in those who sat for 2–3 h, and in students who ate breakfast every day than in those who never or only occasionally ate breakfast. There was no significant difference in the total physical health score according to Internet usage time (*F*(4, 1411) = 1.905, *p* = 0.107, *η*^2^ = 0.005), sleep duration (*F*(3, 1412) = 2.103, *p* = 0.098, *η*^2^ = 0.004), smoking status (*F*(3, 1412) = 1.105, *p* = 0.346, *η*^2^ = 0.002), or alcohol consumption (*F*(3, 1412) = 1.906, *p* = 0.127, *η*^2^ = 0.004).

#### 3.2.2. Comparison of Mental Health Status Among University Student Groups with Different Characteristics

One-way ANOVA was used to analyze differences in UPI total scores across groups defined by categorical variables, including place of origin, family structure, only child status, family financial status, and academic performance. The results showed that there were significant differences in mental health status among university students according to place of origin (*F*(2, 1413) = 3.957, *p* = 0.019, *η*^2^ = 0.006), family economic situation (*F*(3, 1412) = 4.800, *p* = 0.002, *η*^2^ = 0.010), and academic performance (*F*(4, 1411) = 3.429, *p* = 0.008, *η*^2^ = 0.010). Post hoc tests showed that mental health status was better in students from urban or town areas than in those from rural areas. Additionally, students from financially stable families had better mental health status than those from families with relative financial difficulties. The mental health status of university students ranked in the top 20% for academic performance was better than that of those ranked at 20–40%, 40–60% or in the lowest 20%. Furthermore, there was no significant difference in mental health test scores according to family structure (*F*(3, 1412) = 1.808, *p* = 0.144, *η*^2^ = 0.004) or only child status (*F*(1, 1414) = 0.070, *p* = 0.792, *η*^2^ < 0.001).

### 3.3. Correlation Analysis of Factors Associated with Physical and Mental Health in University Students

Relationships between continuous variables (i.e., satisfaction with sports facilities, the family, school, and social exercise atmosphere, and amount of physical exercise) and the total physical health score were examined using Pearson correlation analysis (see Table 1), as were those between continuous variables (i.e., major satisfaction, the quality of a romantic relationship, quality of interpersonal relationships, and family parenting style) and the total mental health score (Table 2). The total physical health score showed a significant positive correlation with the exercise atmosphere at school (*r* = 0.074) and volume of physical exercise (*r* = 0.080). The total mental health score showed a significant negative correlation with satisfaction with academic major (*r* = −0.139), quality of a romantic relationship (*r* = −0.242), quality of interpersonal relationships (*r* = −0.292), an emotionally warm family parenting style (*r* = −0.172). Conversely, it showed a significant positive correlation with both a rejecting (*r* = 0.253) and an overprotective (*r* = 0.254) family parenting style.

### 3.4. Regression Analysis of Factors Associated with Physical and Mental Health in University Students

The regression model for physical health included factors that were significant in the one-way ANOVA (including participation in school sports clubs, sedentary time, and breakfast habits), as well as those significantly correlated with the total physical health test score (i.e., school exercise atmosphere and amount of exercise). Factors that showed a significant difference in one-way ANOVA of mental health scores (i.e., place of origin, family economic situation, and academic performance) and factors that were significantly correlated with the mental health test score (major satisfaction, quality of a romantic relationship, quality of interpersonal relationships, and an emotionally warm, rejecting or overprotective family parenting style) were included in the regression analysis for mental health. Before conducting the hierarchical regression analysis, a multicollinearity test was performed on the independent variables. The variance inflation factor (VIF) for each variable was <3, indicating no serious multicollinearity. After controlling for demographic variables such as sex, academic year, and academic major, the independent variables were entered using the input method (see Table 3 and Table 4). The results showed that participation in school sports clubs (*β* = −0.111, *p* < 0.001) and amount of exercise (*β* = 0.182, *p* < 0.001) were significant predictors of the physical health status of university students. The results also showed that the difficult family economic status (*β* = 0.162, *p* < 0.001), major satisfaction (*β* = −0.092, *p* = 0.020), quality of a romantic relationship (*β* = −0.121, *p* = 0.003), quality of interpersonal relationships (*β* = −0.157, *p* < 0.001), and an overprotective family parenting style (*β* = 0.109, *p* = 0.011) could effectively predict the mental health status of university students.

### 3.5. Machine Learning Analysis of Factors Associated with Physical and Mental Health in University Students

The binary classification of physical and mental health was taken as the target variable. Sex, major, academic year, place of origin, family structure, only child status, family economic situation, academic performance, major satisfaction, quality of a romantic relationship, quality of interpersonal relationships, family parenting style, participation in school sports clubs, Internet usage time, sleep duration, sedentary time, breakfast habits, smoking status, alcohol consumption, satisfaction with sports facilities, the family, school, and social exercise atmosphere, and amount of exercise were used as feature variables. The Naive Bayes, Decision Tree, Random Forest, KNN, XGBoost, and LightGBM models were trained and evaluated for accuracy, precision, recall, F1 score, and the area under the receiver-operating characteristic curve (AUC-ROC).

Comparisons among the models revealed that the Random Forest model achieved the highest AUC-ROC (0.649) and accuracy (0.816) among all evaluated models. The XGBoost model ranked second with an AUC-ROC of 0.621. The Decision Tree and Naive Bayes models achieved AUC-ROC values of 0.612 and 0.606, respectively, representing moderate overall performance. LightGBM achieved an AUC-ROC of 0.589, while the KNN model performed relatively poorly with an AUC-ROC of only 0.554 (Table 5).

**Figure 3:**
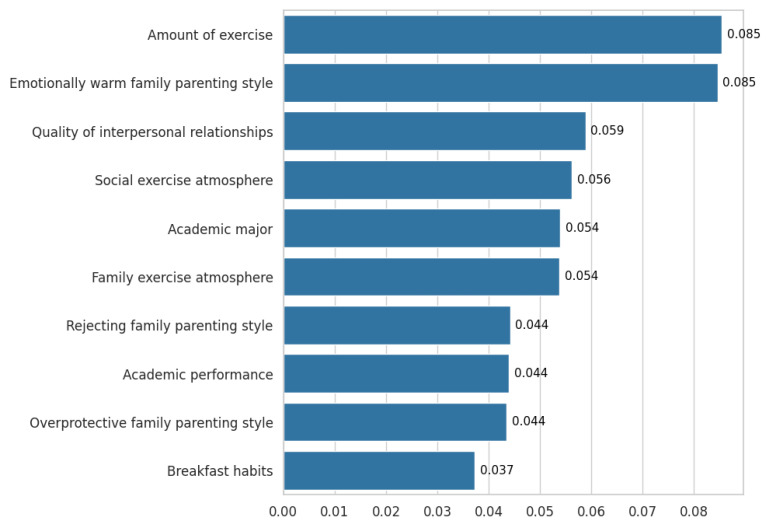
Feature Importance Ranking for Predicting University Students’ Physical and Mental Health Based on the Random Forest Model.

Feature importance analysis (Rengasamy et al., 2022) was performed based on the optimized Random Forest model to complement and extend the findings from traditional statistical analyses. Given the model’s moderate discriminative ability, the results of the feature importance analysis should be interpreted primarily for their exploratory value in ranking predictor relevance, identified the top ten features in relation to university students’ physical and mental health. A higher score indicates a greater relative contribution to the model’s structure. The ranking showed that amount of exercise and emotionally warm family parenting style were assigned the highest importance scores (both 0.085), followed by quality of interpersonal relationships (0.059) and social exercise atmosphere (0.056). Other features, in descending order of relative importance, were academic major (0.054), family exercise atmosphere (0.054), rejecting family parenting style (0.044), academic performance (0.044), overprotective family parenting style (0.044), and breakfast habits (0.037) (Figure 3).

## 4. Discussion

### 4.1. General Characteristics of Physical and Mental Health of University Students

The present study collected and assessed comprehensive physical health and mental health data for the undergraduate population of the 2020 to 2023 cohorts at a university in Guangxi Province of China. This dataset is extensive and provided reliable support for understanding the overall status of physical and mental health in Chinese university students.

The physical health test results showed that 66.9% of university students were in the “acceptable” category, and only 18.2% and 1.6% were in the “good” and “excellent” categories, respectively. This trend might be attributed to the exercise habits and lifestyle patterns prevalent among the university student population. Numerous studies have confirmed that physical exercise is an important factor associated with the physical health of university students (Shores et al., 2015; Eime et al., 2015; Logan et al., 2020), and those with poor lifestyle habits tend to be prone to physical health problems (Rippe, 2018). The results of one-way ANOVA in the subsample revealed that university students who participated in sports clubs and exercised regularly had significantly higher total physical health test scores than those who did not. Moreover, overall physical health test scores were significantly higher in university students who spent less time sedentary and had regular breakfast habits than in those with prolonged sedentary behavior and erratic breakfast habits. Correlation analysis also confirmed a positive relationship between amount of exercise, the exercise atmosphere, and the total physical health test score.

The results of the UPI test showed that 26.3% of the students were at risk of serious psychological problems. The proportion of students at risk was higher than that in the survey of mental health in university students by Fang et al. (2023). These inconsistent findings might reflect the influence of the research methods used. The survey by Fang et al. (2023) only measured the emotional states of depression and anxiety, whereas the UPI used in the present study is a more comprehensive tool for mental health assessment that includes not only emotional states but also multiple aspects such as interpersonal relationships and physical symptoms. Furthermore, the participants in this study were drawn from the western region of China, where the level of economic development is comparatively lower than that in the eastern region. Another reason for the inconsistent results could be the high proportion of students in the western region who come from rural areas. One-way ANOVA in the subsample also revealed that mental health was better in students from urban and town areas than in those from rural areas. Furthermore, mental health status was better among students from financially stable families compared to those from economically disadvantaged families.

### 4.2. Key Factors Associated with University Students’ Physical and Mental Health

The findings of this study are powerfully interpreted through the lens of the biopsychosocial model, which posits that health outcomes arise from the dynamic interplay of biological, psychological, and social factors. Our analysis confirms that the physical and mental health of university students is shaped by a complex network of interrelated factors, often interacting in synergistic ways. When mapped onto the biopsychosocial model, these factors clearly align with its core dimensions. The variables highlighted in this discussion are not based on statistical significance from a single method. Rather, they represent factors that demonstrated consistency across multiple analytical layers: initially identified in preliminary tests (e.g., ANOVA), subsequently confirmed as significant factors in hierarchical regression analyses, and further validated as important features in the machine learning (Random Forest) model. This multi-method convergence strengthens confidence in their role as robust associated factors of student health outcomes. It should be noted that while the identified predictors (e.g., amount of exercise) show significant association with health outcomes within our analytical sample, the specific demographic composition of this subsample (detailed in Section 3.1.2) necessitates caution in directly generalizing these findings to the broader university population. The performance and feature importance of our predictive models are consequently context-specific to the subsample’s profile.

Regression analysis revealed that participation in school sports clubs, amount of exercise, family economic situation, major satisfaction, quality of a romantic relationship, quality of interpersonal relationships, and an overprotective family parenting style could effectively predict the physical or mental health of university students. Meanwhile, the Random Forest model identified the importance of factors such as amount of exercise, emotionally warm family parenting style, quality of interpersonal relationships and social exercise atmosphere, academic major, family exercise atmosphere, rejecting family parenting style, academic performance, overprotective family parenting style, breakfast habits. The differences between the two analytical methods in terms of interpretation of factors associated with physical and mental health might reflect differences in their assumptions, capabilities, and approaches to handling complex data. Integration of the results from regression analysis and machine learning-based assessment identified amount of exercise as a critical correlative factor of the health status of university students. This finding suggests that promotion of participation in physical exercise would be an effective strategy for improvement of physical and mental health in this population. Various analytical methods consistently demonstrated that the quality of interpersonal relationships significantly impacted the physical and mental health of university students, which is in line with the findings reported by Sun (2023). Positive social relationships serve as an important protective factor for the physical and mental health of university students, whereas interpersonal conflict might have negative effects. The effect of family parenting style on physical and mental health was also confirmed by our different analytical methods. This finding aligns with reports in the existing literature (e.g., Brennan et al., 1991), underscoring the consistent and significant role of this factor.

The findings of this study indicate that factors associated with the physical and mental health of university students are primarily related to lifestyle choices and interpersonal interactions. Inadequate participation in physical activity might have a significant negative impact on the physical and mental health of university students. One study of 50,054 Norwegian university students found that physical exercise was negatively associated with mental health problems and suicidality in a dose–response manner (Grasdalsmoen et al., 2020). With the rapid development of the Internet, university students who use mobile phones excessively and are addicted to virtual spaces are less likely to exercise, resulting in a decline in physical health and an increased likelihood of being overweight. Detrimental lifestyle habits are another significant factor impacting the physical and mental health of university students. The competitive pursuit of efficiency and resources within a rapidly evolving society encroach upon students’ already limited time, leading to sedentary behavior, frequent sleep deprivation, and chronic sleep deficiency. These unhealthy habits not only harm university students’ physical health but also make them more prone to negative emotions such as pessimism, anxiety, and burnout, further contributing to various psychological disorders. Furthermore, inappropriate parenting styles significantly impact the physical and mental health of university students. Prolonged parental rejection or overprotection fosters negative psychological perceptions in emerging adults, leading to diminished self-esteem and reduced self-efficacy, which increases the probability of psychological crises. Finally, poor day-to-day interpersonal relationships would adversely impact the physical and mental health of university students. S. Zhang et al. (2023) found that in undergraduate class interpersonal communications, only moderate activity and popularity were positively associated with mental health, suggesting that poor relationships may lack these beneficial associations. University students troubled by interpersonal relationships might intentionally limit their social interactions, weakening their self-efficacy, social skills, and self-confidence, which would have a negative impact on their mental health (Putri et al., 2022). Limiting of external social interactions might also adversely affect physical health by promoting unhealthy habits, such as prolonged Internet use and sedentary behavior. This disruption in lifestyle could compromise the mind–body relationship.

Crucially, our findings underscore the biopsychosocial model’s core tenet: that health correlative factors interact dynamically across domains. For instance, inappropriate family parenting styles such as rejecting family parenting style and overprotective family parenting style can simultaneously constrain psychosocial development and reduce the capacity for self-regulated health behaviors. This illustrates how social-level factors (parenting styles) directly shape both psychological well-being and the behavioral capacity needed to maintain physical health, demonstrating how correlated factors are intertwined across the model’s domains. These cross-domain interactions imply that interventions aimed at enhancing student health must be multifaceted and integrated. They should address not only specific biobehavioral aspects (e.g., exercise) but also the psychological and social systems, such as family dynamics, that shape and sustain those behaviors.

The findings of this study also invite reflection on why some commonly implicated factors, such as sleep duration and general internet usage time, did not emerge as stronger or consistent predictors of physical and mental health in our statistical models. This apparent discrepancy with broader literature may stem from measurement complexity rather than a true lack of association. Firstly, regarding sleep, our study primarily considered sleep duration. However, Bin (2016) underscores that sleep health is multifaceted, encompassing depth, continuity, and subjective restfulness—not just duration. Research specifically on college students indicates that sleep latency (difficulty in falling asleep) may be a more precise predictor of mental health problems than total sleep time alone (Whitehead & Horton, 2025). Therefore, the nuanced aspects of sleep quality, which were not captured in our metrics, might be the critical mediators linking sleep to health outcomes. Secondly, concerning internet use, our analysis included usage time. Yet, the evidence does not support a simple more screen time equals worse mental health narrative. Instead, the type of digital activities and the context in which they occur appear to matter more than the sheer amount of time spent online (Odgers & Jensen, 2020). Future research would benefit from employing more granular and validated measures, such as the Pittsburgh Sleep Quality Index for assessing multidimensional sleep health (Larche et al., 2021) and scales like the Generalized Problematic Internet Use Scale 2 for capturing maladaptive patterns of internet use (Balcerowska & Bereznowski, 2022). This would allow for a more accurate capture of the true influence of these factors on the physical and mental health of university students.

### 4.3. Implications for University Policies and Interventions

The identified key correlative factors point to actionable areas for university-level health promotion. First, regarding physical exercise, universities should move beyond providing facilities to actively cultivate a campus-wide culture of regular activity. This can be achieved by innovating mandatory physical education curricula to include diverse and appealing options, creating strong incentives (e.g., recognition, credits) for participation in sports clubs, and integrating physical activity into daily campus life (e.g., promoting active transportation, offering short exercise breaks during long academic sessions). Second, to address the significant impact of interpersonal relationship quality, student support systems should be strengthened. This includes scaling up structured peer-mentoring programs, offering regular workshops on communication and conflict-resolution skills. Third, acknowledging the influence of family factors, universities can develop more nuanced support mechanisms. Counseling centers can offer workshops or resources on navigating family dynamics and building healthy boundaries, providing support for students experiencing stressful family parenting styles or family conflicts.

The findings of this research should be interpreted in light of several limitations. First, the key predictor analyses relied on a subsample of students who completed the detailed questionnaire. Although this subsample provided the necessary data for examining specific associations, its demographic composition (e.g., overrepresentation of certain majors and underrepresentation of senior students) differs systematically from the overall student population. This limits the generalizability (external validity) of the predictive models and the specific magnitude of effects to the wider university context, although the internal validity of the identified relationships within the subsample remains intact. Second, in the self-designed questionnaire, the use of single-item indicators for key constructs, while necessary for breadth and practicality, may introduce greater measurement error compared to multi-item scales. This error could attenuate the observed effect sizes in our regression and machine learning models, meaning that some true associations may be underestimated. Finally, although the Random Forest model achieved the highest AUC-ROC among all evaluated models, its overall discriminative performance indicates inherent predictive limitations. These limitations are partly attributable to the model’s limited ability to learn discriminative patterns for the minority class, despite the application of SMOTE during training. More fundamentally, physical and mental health outcomes are shaped by a complex interplay of behavioral, environmental, and latent factors—including genetic predispositions, psychological resilience, and a range of other unmeasured variables—that extend beyond the scope of self-reported questionnaire data. Accordingly, the primary contribution of the current machine learning analysis lies in identifying the relative importance of measurable correlated factors and extending the findings from traditional regression analyses, rather than achieving clinical-grade prediction accuracy.

## 5. Conclusions

This study analyzed data for undergraduates from a university with the aim of identifying the core factors associated with physical and mental health in university students. The dataset comprised physical health tests, mental health assessments (using the UPI), and a questionnaire designed to identify related factors. Our analysis, which integrated one-way ANOVA, Pearson correlation, regression analysis, and a machine learning model, found that unhealthy lifestyle habits, such as lack of physical exercise, are detrimental to the physical and mental health of university students. Furthermore, quality of interpersonal relationships and the family parenting style are also associated with the physical and mental health of these students. Cultivating a healthy lifestyle, maintaining harmonious interpersonal relationships, and optimizing family parenting styles play an important role in promoting physical and mental health in the university student population.

## Figures and Tables

**Table 1:** Correlation coefficient matrix for physical health scores in university students and associated factors.

	Total Physical Health Score	Sports Facility Satisfaction	Family Exercise Atmosphere	School Exercise Atmosphere	Social Exercise Atmosphere	Amount of Exercise
Total physical health score	—					
Sports facility satisfaction	0.014	—				
Family exercise atmosphere	0.020	0.136 **	—			
School exercise atmosphere	0.074 **	0.486 **	0.174 **	—		
Social exercise atmosphere	−0.018	0.317 **	0.173 **	0.335 **	—	
Amount of exercise	0.080 **	−0.086 **	0.031	0.049	0.178 **	—

Notes: —indicates duplicate data that are not included. ** *p* < 0.01.

**Table 2:** Correlation coefficient matrix for mental health scores in university students and associated factors.

	UPI Score	Major Satisfaction	Quality of a Romantic Relationship	Quality of Interpersonal Relationships	Emotionally Warm Family Parenting Style	Rejecting Family Parenting Style	Overprotective Parenting Style
UPI score	—						
Major satisfaction	−0.139 **	—					
Quality of a romantic relationship	−0.242 **	0.040	—				
Quality of interpersonal relationships	−0.292 **	0.132 **	0.363 **	—			
Emotionally warm family parenting style	−0.172 **	0.227 **	0.102 *	0.159 **	—		
Rejecting family parenting style	0.253 **	−0.123 **	−0.273 **	−0.352 **	−0.359 **	—	
Overprotective parenting style	0.254 **	−0.058 *	−0.219 **	−0.264 **	−0.099 **	0.430 **	—

Notes: —indicates duplicate data that are not included. * *p* < 0.05, ** *p* < 0.01. UPI, University Personality Inventory.

**Table 3:** Multiple linear regression analysis of factors associated with the total physical health score in university students.

Variable	Total Physical Health Test Score					
	B	95%CI	SE	*β*	t	*p*
Constant	70.041	(66.251–73.831)	1.932	—	36.251	<0.001
Sex (with “male” as reference)	7.188	(6.216–8.159)	0.495	0.394	14.520	<0.001
Academic major (with “Humanities and Social Sciences “ as reference)	—	—	—	—	—	—
Science and Engineering	0.653	(−0.790–2.095)	0.735	0.023	0.888	0.375
Arts and Physical Education	−1.222	(−7.157–4.714)	3.026	−0.010	−0.404	0.687
Academic year (Taking “Freshman” as a reference)	—	—	—	—	—	—
Sophomore	2.064	(1.033–3.094)	0.525	0.105	3.930	<0.001
Junior	−0.376	(−1.486–0.733)	0.565	−0.018	−0.666	0.506
Senior	−2.125	(−6.535–2.285)	2.248	−0.023	−0.945	0.345
Participation in school sports clubs (with “Participating in school sports clubs” as reference)	—		—	—	—	—
Not participating in school sports clubs	−2.804	(−4.038–−1.570)	0.629	−0.111	−4.459	<0.001
Sedentary time (with “Less than 1 h” as reference)	—	—	—	—	—	—
2–3 h	−1.368	(−4.041–1.305)	1.362	−0.060	−1.004	0.315
3–4 h	0.087	(−2.542–2.716)	1.340	0.004	0.065	0.948
Longer than 5 h	−0.126	(−2.692–2.439)	1.308	−0.007	−0.097	0.923
Breakfast habits (with “Never” as reference)	—	—	—	—	—	—
Occasionally	−0.134	(−2.200–1.933)	1.053	−0.007	−0.127	0.899
Frequently	0.516	(−1.568–2.599)	1.062	0.027	0.486	0.627
Every day	1.092	(−1.133–3.317)	1.134	0.046	0.963	0.336
School exercise atmosphere	−0.021	(−0.544–0.502)	0.267	−0.002	−0.080	0.936
Amount of exercise	0.097	(0.069–0.124)	0.014	0.182	6.912	<0.001

Notes: —indicates that these data are unavailable. The control variables were sex, major, and academic year. CI, confidence interval; SE, standard error.

**Table 4:** Multiple linear regression analysis of factors associated with mental health in university students.

Variable	Total Score of Mental Health Test Score (UPI Score)					
	B	95%CI	SE	*β*	t	*p*
Constant	23.874	(9.387~15.802)	4.124	—	5.789	<0.001
Sex (with “male” as reference)	1.334	(−0.351–3.019)	0.858	0.062	1.555	0.121
Academic major (with “Humanities and Social Sciences” as reference)	—	—	—	—	—	—
Science and Engineering	−4.234	(−6.998–−1.470)	1.407	−0.122	−3.009	0.003
Arts and Physical Education	−2.322	(−12.212–7.569)	5.035	−0.018	−0.461	0.645
Academic year (with “Freshman” as a reference)	—	—	—	—	—	—
Sophomore	1.086	(−0.761–2.933)	0.940	0.048	1.155	0.249
Junior	1.903	(−0.088–3.895)	1.014	0.078	1.877	0.061
Senior	−0.624	(−8.537–7.288)	4.028	−0.006	−0.155	0.877
Place of origin (with “Urban areas” as reference)	—	—	—	—	—	—
Town areas	0.183	(−2.193–2.559)	1.21	0.007	0.151	0.880
Rural areas	1.141	(−1.024–3.306)	1.102	0.054	1.035	0.301
Family economic situation (with “Financially stable” as reference)	—	—	—	—	—	—
Facing financial difficulties	0.806	(−1.208–2.821)	1.026	0.033	0.786	0.432
Experiencing financial hardship	4.554	(2.226–6.883)	1.185	0.162	3.842	<0.001
Severe financial hardship	1.140	(−1.961–4.241)	1.579	0.030	0.722	0.471
Academic performance (with top “20%” as reference)	—	—	—	—	—	—
20–40%	0.643	(−1.589–2.875)	1.137	0.025	0.566	0.572
40–60%	0.938	(−1.207–3.082)	1.092	0.039	0.859	0.391
60–80%	1.641	(−0.878–4.161)	1.283	0.055	1.28	0.201
Lowest 20%	2.277	(−0.702–5.257)	1.517	0.063	1.501	0.134
Major satisfaction	−1.244	(−2.296–−0.193)	0.535	−0.092	−2.324	0.020
Quality of a romantic relationship	−1.401	(−2.339–−0.464)	0.477	−0.121	−2.935	0.003
Quality of interpersonal relationships	−1.903	(−2.909–−0.896)	0.512	−0.157	−3.713	<0.001
Emotionally warm family parenting style	−0.343	(−1.163–0.476)	0.417	−0.034	−0.823	0.411
Rejecting family parenting style	0.957	(−0.101–2.015)	0.538	0.082	1.777	0.076
Overprotective family parenting style	1.094	(0.254–1.935)	0.428	0.109	2.557	0.011

Notes: —indicates that these data are unavailable. The control variables were sex, major, and academic year. CI, confidence interval; SE, standard error; UPI, University Personality Inventory.

**Table 5:** Comparison of performance of models for determining physical and mental health in university students.

Model	Accuracy	Precision	Recall	F1 Score	AUC-ROC
Naive Bayes	0.604	0.237	0.532	0.328	0.606
Decision Tree	0.726	0.281	0.328	0.301	0.612
Random Forest	0.816	0.474	0.117	0.188	0.649
KNN	0.682	0.221	0.299	0.254	0.554
XGBoost	0.769	0.322	0.247	0.279	0.621
LightGBM	0.790	0.370	0.221	0.276	0.589

## Data Availability

The data presented in this study are available upon request from the corresponding author due to privacy restrictions.

## Abbreviations

The following abbreviations are used in this manuscript:

ANOVA	One-way analysis of variance
UPI	University Personality Inventory
AUC-ROC	area under the receiver-operating characteristic curve
